# The effects of the cognitive bias of unrealistic optimism in the adoption of preventive measures against COVID-19 in dentistry

**DOI:** 10.47626/1679-4435-2022-834

**Published:** 2022-03-30

**Authors:** Allana Pivovar, Elder Semprebon, Nicole Nichele Perdoncini, Amanda Rossi Corelhano, Cassius Carvalho Torres-Pereira

**Affiliations:** 1 Programa de Pós-Graduação em Odontologia, Universidade Federal do Paraná (UFPR), Curitiba, PR, Brazil.; 2 Escola de Administração, UFPR, Curitiba, PR, Brazil.

**Keywords:** dentistry, COVID-19, SARS-CoV-2, optimism, bias

## Abstract

**Introduction:**

The bias of unrealistic optimism is people’s tendency to believe that they are less likely to experience negative events than others. Dental professionals are part of a high-risk group for COVID-19.

**Objectives:**

The objective of this study is to investigate whether unrealistic optimism bias, concern, and regret can affect the adoption of preventive measures by these professionals.

**Methods:**

We conducted a cross-sectional observational study of dentists recruited remotely by using an electronic form sent by email and social media. A five-point scale was used to identify whether unrealistic optimism, concern, and regret affected the adoption of preventive measures. The study included a total of 339 dentists. The group considered to be unrealistically optimistic accounted for 24.8% of the sample.

**Results:**

Unrealistic optimism negatively affected the use of protective equipment by dental professionals. Conversely, concern positively affected preventive measures.

**Conclusions:**

Although most dentists are realistic about the risk of infection compared with their peers, those with optimistic bias might be more susceptible to the disease, as they tend not to adopt recommended protective measures in the workplace. Future studies should also investigate ways of debiasing.

## Introduction

The recent spread of the novel severe acute respiratory syndrome coronavirus 2 (SARS-CoV-2) has affected the entire international community and caused wide-ranging public health problems.^1^ Given the widespread transmission of SARS-CoV-2 and reports of its spread to health care providers, dental professionals are at high risk of infection and may become potential carriers of the disease. These risks can be attributed to the invasive nature of dental interventions, which include aerosol generation, handling of sharps, and proximity of the dental professional to the patient’s oropharyngeal region. In addition, if proper precautions are not taken, the dental office can potentially expose patients to cross-contamination.^1,2^ Infection control measures are needed to prevent the virus from spreading and help control the pandemic situation.^2^

Individuals must make rational choices for their own benefit, but they are not always rational actors.^3^ Cognitive biases occur when acquiring and processing information, filtering it through personal beliefs and experiences. Flaws in judgment happen due to false memories, social attributions, or statistical errors.^4^ People develop cognitive biases for many reasons, but they are often the result of a system of heuristic processes (problem-solving strategies) that help the brain process information quickly. Although heuristics are important and useful in solving problems in everyday life, under conditions of complexity and uncertainty, they can produce systematic errors in judgment.^4^ These predictable errors can be observed in the areas of analysis of intelligence, politics, law, health, education, business, and private life.^4^

The bias of optimism or the bias of unrealistic optimism is people’s tendency to believe that they are less likely to experience negative events and more likely to experience positive events than others.^5^ This belief has been demonstrated in several domains including breast cancer, traffic accidents, HIV, heart attacks, and natural disasters.^6^ Regarding negative events, the perception that one is comparatively less vulnerable than others has been associated with reductions in worry, intentions, and behaviors associated with self-protection and prevention.^6^ This bias can also explain a series of harmful health behaviors. Greater unrealistic optimism about avoidance of negative problems with alcohol corresponded to greater alcohol consumption a year later,^7^ as well as greater unrealistic optimism about how to avoid H1N1 influenza corresponded to lower intentions to engage in practices of hand hygiene.^8^ Therefore, unrealistic optimism can have important and harmful consequences for people’s future.

Affect also explicitly identifies affective feelings as integral components of risk beliefs,^9^ and these feelings are believed to consist of viscerally experienced and anticipated emotions.^9^ Examples include concern or anxiety (emotions that generate an immediate visceral response) and regret or disappointment (anticipated emotions, which are expected to be experienced in the future).^10^ Affective probability is about how people report feelings concerning their risk, rather than their judgments of cognitive probability. Concern (i.e., people’s concerns about a specific risk) and anticipated regret (i.e., how sorry people would feel when imagining that an illness would occur because of their risky behavior) were associated with several health-related behaviors, such as mammography screening, influenza vaccination, sunscreen use, smoking cessation, and colon cancer screening intentions.^10^

It is the responsibility of dentists to adopt infection prevention and control measures to prevent or reduce the transmission of microorganisms as much as possible during any dental care performed in their office. The objective of this study is to investigate whether cognitive and affective factors, such as unrealistic optimism bias, concern, and regret, can affect the adoption of preventive measures by dental professionals, who are a high-risk group in the context of the COVID-19 pandemic.

## Methods

### Procedures

This cross-sectional observational study was approved by the research ethics committee of Universidade Federal do Paraná (CAAE 35238620.1.0000.0102). A convenience sample was recruited remotely through snowball sampling by sending a standard message to an email list provided by the Regional Board of Dentistry of the state of Paraná (Brazil), which contains nearly 23,000 email signatures. Dentists working with patient care in dental services from March 2020 until the time of the study were invited to participate even if there was a reduction in the number of hours in a workday or a partial suspension of activities during this period. Exclusion criteria were presence of a confirmed diagnosis of COVID-19 and total suspension of all activities since March 2020 due to the COVID-19 pandemic or due to other personal reasons.

Data were collected from July to December 2020 using an electronic form without participant identification. The questionnaires were completed by the participants remotely and independently of the researcher. All respondents were debriefed upon completion of the questionnaire.

### Instruments

The questionnaire had an introduction that briefly explained the purposes of the study in a way that the real objective of the study was not revealed, thus not influencing the responses. After reading the explanation, the respondents were required to provide written informed consent. The questionnaire consisted of 38 questions divided into five sections. The first section had questions related to the risk of infection (six questions). The second section had questions related to the characteristics of the performance in the dental service market (five questions). The third section had questions related to preventive measures towards COVID-19 in clinical dental practice (18 questions). These measures were obtained from the Manual of Good Biosafety Practices for Dental Environments released with institutional support from the Brazilian Federal Board of Dentistry and scientific support from the Latin American Institute of Dental Research and Education (Instituto Latino Americano de Pesquisa e Ensino Odontológico, ILAPEO) and the International Team for Implantology (ITI).^11^ The fourth section collected sociodemographic data (six questions). Finally, the last section had questions to assess the understanding of the research statements (three questions).

Unrealistic optimism was measured by asking a question adapted from Bränström et al.^12^: “How likely are you to contract COVID-19 compared with other dentists the same age as you?”, rated on a five-point scale (1 - Much less likely than other people and 5 - Much more likely than other people).

Concern was assessed by asking a question adapted from Janssen et al.^10^: “How concerned are you about contracting COVID-19?”, rated on a five-point scale (1 - Not at all and 5 - Extremely concerned).

Finally, regret was assessed by asking a question adapted from Ziarnowski et al.^13^ and Janssen et al.^10^: “How much regret would you feel if you were to fall ill from COVID-19 in the near future because of your work?”, rated on a five-point scale (1 - Little regret and 5 - Much regret).

### Data analysis

The data collected through the electronic forms were entered in an electronic spreadsheet and later analyzed with IBM SPSS Statistics (version 20).

## Results

A total of 339 dentists participated in the survey. Most participants were women (n = 246; 72%) and worked in the private sector (n = 243; 71.7%), with 13.9% working in the public sector, and 15.5% in both. In addition, 88.8% (n =301) self-identified as white and had graduate training. Few participants had one or more conditions considered to be at risk for COVID-19, such as obesity, hypertension, diabetes, immunosuppression, and breathing problems (n = 74; 21.8%).

On a five-point scale, participants considered the research relevant (mean [M] = 4.58; standard deviation [SD] = 0.739), had no difficulty in responding it (M = 4.51; SD = 0.927) and were committed to their participation (M = 4.59; SD = 0.994).

### Unrealistic optimism

The group considered to be unrealistically optimistic accounted for 24.8% (n = 84) of the sample, as the participants reported that their likelihood of contracting COVID-19 was lower (18.6%, n = 63) or much lower (6.2%, n = 21) than that of other dental professionals. As for the other respondents, 61.9% (n = 210) were considered realistic, as they answered that they had the same chance, and 13.3% (n = 45) were considered pessimistic, since they answered that their likelihood of contracting COVID-19 was higher (11.8%, n=40) or much higher (1.5%, n = 5).

Linear regression was used to determine the influence of unrealistic optimism on the adoption of preventive measures by dental professionals. The results are shown in Table 1 and indicate that unrealistic optimism negatively affected the use of protective equipment by dental professionals, such as the use of N95 or PFF2 masks (β =-0.218; p = 0.021), face shields (β =-0.187; p = 0.028), overcoat or disposable coveralls (β =-0.262; p = 0.014), and double gloves to handle non-sterilizable devices (β =-0.233; p = 0.048). In addition, unrealistic optimism also negatively affected preventive practices, such as daily installation of disinfectant mats or use of disposable shoe covers for patients (β =-0.393; p = 0.002), sterilization of all handpieces (high and low speed) (β =-0.250; p = 0.026), and longer scheduling intervals between patients (β =-0.279; p = 0.003).

**Table 1:** Linear regression of the influence of unrealistic optimism on the adoption of preventive measures by dental care professionals

Preventive measure	Mean	SD	β	SE (β)	t	p-value
N95 or PFF2 mask	4.124	1.333	-0.218	0.094	-2.316	0.021
Face shield	4.074	1.198	-0.187	0.085	-2.203	0.028
Disposable overcoat / coveralls	3.602	1.503	-0.262	0.106	-2.473	0.014
Surgical scrubs, uniform, or specific clothing for the work environment	3.204	1.658	-0.009	0.118	-0.077	0.938
Double gloves to handle non-sterilizable devices	2.853	1.659	-0.233	0.117	-1.983	0.048
Rapid tests for the detection of COVID-19	1.584	1.175	0.132	0.083	1.584	0.114
Daily installation of disinfectant mats or use of disposable shoe covers for patients	3.112	1.822	-0.393	0.128	-3.073	0.002
Hand washbasins and alcohol-based hand sanitizers for patient use	4.617	0.850	-0.066	0.060	-1.095	0.274
Chairs placed 1 m apart in the waiting room	4.516	0.927	-0.079	0.066	-1.197	0.232
An aerosol suction device connected to the vacuum pump	2.204	1.841	-0.191	0.131	-1.462	0.145
Sterilization of all handpieces (high and low speed)	3.209	1.577	-0.250	0.111	-2.243	0.026
Measurement of the patient’s body temperature	2.729	1.760	0.051	0.125	0.406	0.685
Use of pre-service antimicrobial mouthwash	3.425	1.587	-0.129	0.113	-1.149	0.252
Longer scheduling intervals between patients	3.850	1.323	-0.279	0.093	-3.004	0.003
Urgent and emergency care only	2.218	1.271	-0.020	0.090	-0.223	0.824
Temporary interruption of activities	2.507	1.193	-0.106	0.085	-1.258	0.209
Increase in the prices and fees of the services provided	1.761	1.307	-0.001	0.093	-0.008	0.994
Increased virtual interaction with patients (phone calls, WhatsApp conversations, virtual consultations for initial assessment, and telemonitoring or tele-guidance)	2.714	1.436	-0.079	0.102	-0.778	0.437
Preventive measures - General	3.128	0.714	-0.129	0.050	-2.561	0.011

SD = standard deviation; SE = standard error.

### Concern and regret

Unrealistic optimism was not associated with feelings of concern (p > 0.05) or regret (p > 0.05). Of these feelings, concern was found to positively affect preventive measures (general mean score) (β = 0.146; p = 0.001), whereas regret had no association with these measures (p > 0.05)

## Discussion

The present study showed that the cognitive bias of unrealistic optimism in dentists may affect preventive behaviors against COVID-19 in this sample. Although most dentists are realistic about the risk of infection compared with their peers, those with unrealistic optimism bias might be more susceptible to the disease, as they do not adopt recommended protective measures in the workplace. In addition to putting themselves at risk, these dental professionals potentiate the risk of cross-contamination of patients. They are not only potential transmitters but also vulnerable during dental care. Moreover, other involved professionals, such as assistants, secretaries, and cleaning personnel, are also exposed due to the dentist’s recklessness.

### Perceived risk, optimistic bias, and COVID-19

Perceived susceptibility to the disease or perceived risk is a major determinant of prevention. In the context of the COVID-19 pandemic, engagement in preventive measures was strongly predicted by the perceived likelihood of personally being infected: the higher their perceived likelihood, the more people engaged in protective behaviors.^14^

However, by decreasing the perceived risk, optimistic bias can undermine an individual’s motivation to take precautions. Park et al.^15^ recruited 293 U.S. adults using online survey platforms in order to determine the relationship between optimistic bias and preventive behavioral engagement in the context of COVID-19. Optimistically biased respondents perceived their risk of COVID-19 to be low. Also, perceived risk was related positively to affective responses to risk (e.g., worry and fear). That is, the lower their perceived risk of COVID-19, the less likely respondents were to feel anxiety and fear about this disease.^15^

It appears that unrealistic optimism has played a key role in individual beliefs about the consequences of the pandemic itself. Citizens in the United States, Europe, and the United Kingdom estimated the likelihood of being infected with the virus and subsequently infecting others as lower for themselves than for others.^16,17^ Also, a study in Romania and Italy showed that optimism bias in the context of COVID-19 was present in both countries and concluded that it depends on self-reported health status and increases with age.^18^ Therefore, optimistic bias is a consistent phenomenon occurring in the COVID-19 pandemic.

### Unrealistic optimism, dentistry, and emotions

In the dental care setting, dentists are at high risk for COVID-19. However, due to the effectiveness of personal protective equipment (PPE), the risk of COVID-19 transmission in dental offices is low.^19^ A six-month longitudinal study of dentists in the United States showed a COVID-19 infection prevalence rate of 2.6% and incidence rates ranging from 0.2% to 1.1% each month.^20^ Yet, the lack of reported COVID-19 infections among dental health care personnel should not be taken as evidence for low or negligible risk.^21^ Conway et al.^22^ reported a COVID-19 testing survey of asymptomatic-screened patients presenting in a dental setting and showed that 0.5% (95% confidence interval [95%CI] 0.5-0.8) tested positive for SARS-CoV-2. In addition, the positivity rate in this patient group reflected the underlying prevalence in the community at the time.^22^ The need for combined biosafety efforts and measures is fundamental to ensure safety in the dental care setting.^20^ In a multinational study, dentists were found to obtain good knowledge and practice scores about COVID-19.^23^ It appears that simply understanding information about risk factors may not be sufficient for people to formulate a judgment of their own vulnerability to harm.^12^

Regarding affective feelings, the results of the present study suggest that concern is positively related to the adoption of preventive measures. If individuals perceive risk, affective responses to risk arise—and these emotional responses motivate individuals to seek information that will help reduce the risk.^15^ De Stefani et al.^24^ used a questionnaire to evaluate Italian dentists’ perceptions and attitudes regarding COVID-19 infection from April 11 to 18, 2020, more than a month after the outbreak in Italy. Dentists considered the virus infection highly dangerous and were not confident in being able to work safely.^24^ Also, Brazilian dentists demonstrated fear of contracting the disease at work, in association with regional COVID-19 incidence/mortality.^25^ In fact, affective probability judgments have been found to be stronger predictors of behavior than cognitive probability judgments for involvement in sunscreen use,^26^ for obtaining influenza vaccination,^27^ for the intention to participate in colon cancer screening,^28^ and for the intention to stop smoking.^10^ Because our survey was observational, we were unable to analyze how different national phenomena could be influencing the affective probabilities, but we can assume that there is probably a strong influence.

Regret did not have an effect on optimistic bias or adoption of preventive measures. Messages that make risk very real, by increasing perceived vulnerability, can be effective in some conditions, but they can also backfire. While positive emotions can make amplified health risks acceptable, negative emotions seem to lead to the rejection of risk-promoting messages. Similarly, the literature on fear appeals indicates that appeals that create fear or stress can be effective only up to a certain limit.^29^ This may explain why regret did not appear to be relevant in the present analysis.

Park et al.^15^ suggest that the actual risk of COVID-19 should be reinforced in order to reduce optimism bias. However, maintaining individual risk perceptions at a relatively high level despite the prolongation of the crisis can be challenging. As observed during the H1N1 pandemic, prolonged exposure to threat increases feelings of familiarity, progressively reducing perceived risk.^30^

## Conclusions

This study is a pioneer in evaluating unrealistic optimism in the context of the COVID-19 pandemic among dentists. However, the small sample size did not allow us to perform a sociodemographic analysis to better understand the factors that may be influencing this phenomenon, such as years of experience, sex, age, and specialization. Also, data were collected based on a convenience sampling technique combined with snowball sampling. Although there is support in the literature that the sample size eliminated the risk of bias, it should be considered that a similar study would bring more information if conducted with country-representative data. Regarding the low adherence rate in the recruitment strategy via email list, a possible explanation is that there is an email overload related to the pandemic in the network—what does not mean that communication is necessarily being effective. Communication from public authorities plays a key role in this scenario to provide appropriate information in order to reduce psychological effects.^18^

Although most dentists are realistic about the risk of infection compared with their peers, those with unrealistic optimistic bias might be more susceptible to the disease and expose patients to cross-contamination, as they do not adopt recommended protective measures in the workplace. The identification of this bias can help prepare for future waves of disease outbreaks and early adoption of precautionary measures. Future studies should also investigate ways of debiasing, which may help improve adherence to protective measures among dental professionals.

### Data availability statement

The data that support the findings of this study are available from the corresponding author, upon reasonable request.
